# Treatment of pediatric fistula-in-ano—Sphincter-sparing non-cutting seton placement as the future treatment of choice?

**DOI:** 10.3389/fsurg.2023.1144425

**Published:** 2023-04-11

**Authors:** Manuel Besendörfer, Laurin Langer, Roman Carbon, Christel Weiss, Hanna Müller, Sonja Diez

**Affiliations:** ^1^ Friedrich-Alexander-Universität (FAU) Erlangen-Nürnberg, Department of Surgery, Section of Pediatric Surgery, University Hospital Erlangen, Erlangen, Germany; ^2^ Department of Medical Statistics, Biomathematics, and Information Processing, Medical Faculty Mannheim of Heidelberg University, Mannheim, Germany; ^3^ Division of Neonatology and Pediatric Intensive Care, Department of Pediatrics, University Hospital Marburg, University of Marburg, Marburg, Germany

**Keywords:** pediatric fistula-in-ano, perianal abscess, placement of seton, loose seton, sphincter sparing

## Abstract

**Background:**

Therapeutic principles of fistula-in-ano (FIA) are lacking evidence-based consensus on treatment options. Non-cutting, sphincter-sparing options have not been published for infancy and childhood FIA.

**Patients and methods:**

We are presenting retrospective data on FIA treatment with non-cutting seton placement between 2011 and 2020. Data were collected based on medical records and complemented by patients’ contact for follow-up analyses between November 2021 and October 2022. Data were analyzed regarding the outcome variables of recurrent FIA and recurrent perianal abscess. Furthermore, outcomes in different age groups were compared (<1/1.5–12 years of age).

**Results:**

Treatment duration with non-cutting seton was at a median of 4.6 months and was not associated with recurrent FIA (*p* = 0.8893). Overall recurrence rate of FIA within an observation time of 9 months postsurgically was at 7% (*n* = 3/42) and was only seen in infancy, whereas recurrent perianal abscess was mainly observable in children (*n* = 2, *p* = 0.2132). Comparison of age groups revealed no significant differences. Of the 42 included patients, 37 responded in the follow-up analysis, resulting in a response rate of 88% with a median follow-up time of 4.9 years. Fecal incontinence was postsurgically only seen in two patients, who were diagnosed prior to surgery and symptoms remained unchanged.

**Conclusions:**

Non-cutting seton placement might be a promising option in the treatment of FIA in infancy and childhood. Perioperative settings like duration of placed seton and antibiotic treatment have to be discussed in further prospective, enlarged population-based studies.

## 1. Introduction

Therapy for fistula-in-ano (FIA) in infancy and childhood is lacking evidence-based options and consensus on general guidelines. Individual treatment relies therefore on the empiric decision of the attending surgeon and is based on principles of adult experiences. However, classical adult approaches like fistulotomy or seton placement were expanded by the implementation of sphincter-sparing techniques, e.g., non-cutting seton, dermal flaps, or fibrin sealant injections (1), which have not yet been transferred to pediatric surgery despite a singular case presentation of non-cutting seton placement in children >1.5 years of age (2).

Emile et al. presented in 2016 the singular systematic review on the treatment of FIA in infants (3). Even if conservative approaches are discussed with promising results (4), surgical options are revealed to be significantly preferred in this review. Out of 399 surgically treated infants (of 490 included patients), 65% received fistulotomy, 21% fistulectomy, and 12% placement of a cutting seton. The summary of postsurgical complications confirmed favorable results for one concept: placement of a cutting seton was associated with a low recurrence rate (4% vs. 7% in fistulotomy vs. 1% in fistulectomy) and showed no complications (vs. 9% in fistulectomy vs. 1% in fistulotomy). However, long-term follow-up was not provided and perioperative therapeutic settings, such as antibiotic treatment or duration of placed seton, were not discussed. Furthermore, techniques of non-cutting seton placement were not evaluated and are rarely published so far.

We have treated patients in our specialized center with seton placement since 2011. Based on the adaption of adult coloproctological principles, we applied a non-cutting seton without surgical revisions as a sphincter-sparing technique, which supports drainage, granulation, and epithelialization of the fistulous tract. We here present the outcome of non-cutting seton placement in pediatric patients with FIA in our institution retrospectively with the goal to illustrate follow-up data of this method in infancy.

## 2. Patients and methods

### 2.1. Data management

This is a retrospective analysis of patients with FIA, who were treated surgically within January 2011 and December 2020 in our institution. All children presenting with an intra- or transsphincteric FIA according to the Park classification (5), which includes the majority of the sphincter complex, were included in the study. Patients with inflammatory bowel disease were excluded from further analyses.

Patients’ preoperative characteristics, surgical data, and clinical outcomes were compiled from medical records of routine perioperative check-ups. Additionally, patients were contacted by telephone to obtain further follow-up data between November 2021 and October 2022. At these contacts, informed consent was obtained for participation from each patient and data collection was based on a specialized questionnaire. This questionnaire was developed to classify possible problems of wound healing and especially sequela (defecation pain, anal fissures, constipation, anal bleeding, fecal incontinence, perianal dermatitis). If any of the questions affirmed a problem, patient's direct contact to the outpatient clinic was established and further therapy was introduced. The study was conducted based on the Declaration of Helsinki and further amendments and was furthermore approved by the local ethics committee (No. 20–240_1-B).

### 2.2. Surgical technique

**Figure 1:**
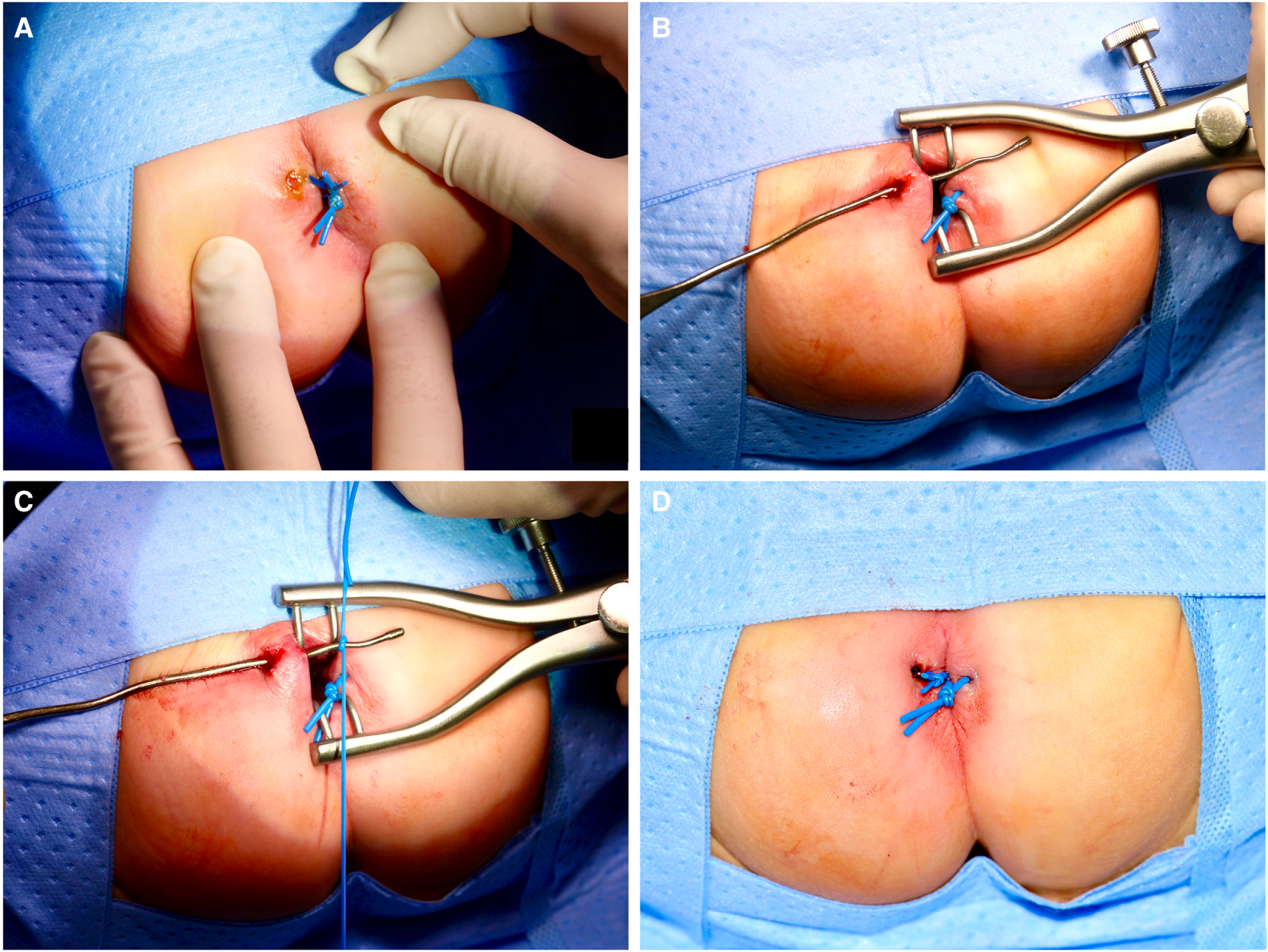
The surgical technique is illustrated by an exemplary case of an infant of 10 months of age with two FIA. After placement of seton in a FIA at 3 o’clock, FIA with concomitant perianal abscess at 10 o’clock is explored (**A**). FIA is identified by the insertion of a bulb-headed lacrimal probe (**B**). The silicone vessel loop is attached (**C**) and pulled trough. The loop is ligated loosely outside the anus without any additional tension to the skin (**D**).

Silicon seton placement was conducted under general anesthesia. Identification of the FIA was secured by the insertion of a bulb-headed lacrimal probe, on which a silicon vessel loop was attached and pulled through the entire fistulous tract (see Figures 1A–C). In case of a perianal abscess, drainage and sphincter-sparing debridement was performed followed by secondary wound healing. The loop was ligated loosely outside the anus without any additional tension to the skin (see Figure 1D). Regular surgical revision of the seton was waived. In routine follow-ups, it was removed if it did not fall out spontaneously. Time point of removal was set individually: decision was made by the attending surgeon based on clinical findings without any signs of inflammation or purulent secretion at the FIA for at least 6 weeks.

### 2.3. Statistical analyses

Analyses were made based on primary outcome variables, defined as recurrent FIA or recurrent perianal abscess. Patients were divided into two study groups according to the patient's age at diagnosis (group 1: infants between 0 and 12 months of age, group 2: children >1 year of age). Data were statistically analyzed using the SAS software (release 9.4; SAS Institute Inc., Cary, NC, United States). Quantitative variables were summarized as median with interquartile range (IQR). Categorical variables were summarized as percentages. Continuous variables were compared using the nonparametric Mann–Whitney *U*-test. Fisher's exact test and Chi square test were used for comparing categorical data. Differences were considered significant at *p* < 0.05.

## 3. Results

**Figure 2:**
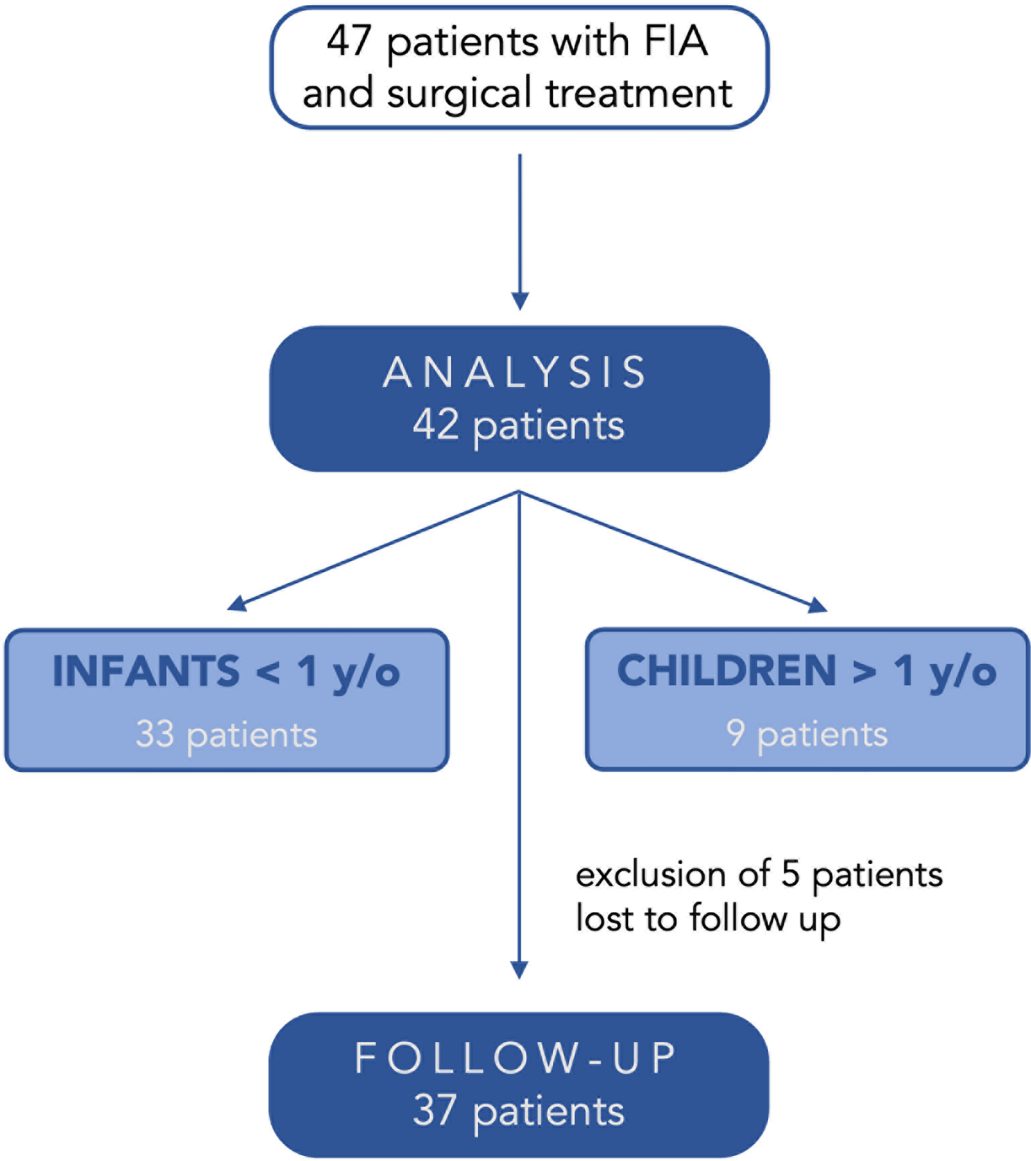
The study design.

Within the study period of 10 years, 47 patients were treated due to FIA in our specialized center. No patient received conservative treatment. After exclusion of 5 patients with inflammatory bowel disease, a total of 42 patients were included in further analyses and were contacted to collect follow-up data. Of these, 37 patients responded to our inquiry, resulting in a response rate of 88%. Median follow-up time was 4 years and 11 months. The study's design is depicted in Figure 2.

Patients’ demographic baseline data are shown in Table 1. We included patients with secondary diagnoses despite inflammatory bowel diseases. In two cases, syndromes were identified without association of inflammatory intestinal processes. Prior to diagnosis and seton placement at our institution, four infants were diagnosed with FIA and treated with fistulectomy (*n* = 2) and cutting seton placement (*n* = 2) in other children's hospitals. These patients were also included in our study.

**Table 1:** Patients’ demographical characteristics.

	All participants (*n* = 42)
**Age at diagnosis [median in months (range)]**	6 (1–146)
**Sex**	
Male	41 (98%)
Female	1 (2%)
**Secondary diagnoses**	
Cardiac	4 (9%)
Gastrointestinal	2 (5%)
Syndromic	2 (5%)
Urogenital	3 (7%)
None	31 (74%)
**FIA without perianal abscess at the timepoint of FIA diagnosis**	
Yes	7 (17%)
No	35 (83%)
**Surgery for perianal abscess prior to FIA identification**	
Yes	17 (40%)
No	25 (60%)

FIA, fistula-in-ano.

Every patient received placement of a non-cutting seton as described above. Duration of the placed seton was at a median of 4.6 months (range 2 days to 11.0 months) and was not associated with the occurrence of recurrent FIA or perianal abscess (*p* = 0.8893). Overall, FIA recurrence rate was at 7% (3/42) within an observational time of 9 months postsurgically: in two patients, an FIA occurred at 1.0 and 3.5 months while the seton was still in place, which made surgical revision necessary. One patient suffered from a recurrent FIA 1.0 months after removal of the seton (2%, *n* = 1/42). Recurrent perianal abscess occurred in three patients within 3 months after seton placement. In no patient, revision of seton placement was necessary. Data on long-term sequela were collected based on the questionnaire and included perianal dermatitis, constipation, and fecal incontinence, as indicated by the parents in 6/37 patients (16%). Perianal dermatitis was seen after full recovery of the FIA treatment in two patients (5%), and fecal incontinence only occurred in children suffering from this condition prior to FIA surgery.

Results of outcome comparing age groups of infants (up to 12 months of age) and children (1–12 years) are presented in Table 2. There were no significant differences regarding presurgical setting and surgical outcome. Duration and removal of seton was equal in the comparison of the age groups. Although recurrent FIA was only observable in infants (9%, *n* = 3), recurrent abscess was mainly seen in older children (22%, *p* = 0.2132). No long-term sequela was significantly associated with age groups or outcome variables. Increasing incidence of any postsurgical condition was associated with increasing age though (*p* = 0.0315).

**Table 2:** Surgical characteristics and outcome concerning age groups (infants < 12 months vs. children > 12 months).

	Infants (*n* = 33)	Children (*n* = 9)	*p*-value
**Age at diagnosis [median in months (range)]**	4 (1–12)	31 (18–146)	<0.0001
**Surgery for perianal abscess prior to FIA identification**			0.2710
Yes	15 (45%)	2 (22%)	
No	18 (55%)	7 (78%)	
**Perioperative antibiotic treatment**			1.000
Yes	13 (39%)	3 (33%)	
No	20 (61%)	6 (67%)	
**Duration of seton [median in days (range)]**	145 (20–329)	134 (2–210)	0.4257
**Removal of seton**			0.3213
By chance	6 (18%)	2 (22%)	
By attending physician	19 (58%)	7 (78%)	
Unknown	8 (24%)	0	
**Postsurgical complications**			0.2132
None	29 (88%)	7 (78%)	
Perianal abscess	1 (3%)	2 (22%)	
Re-FIA	3 (9%)	0	
**Long-term sequela**	*n *=* *28	*n *=* *9	0.2884
Constipation	1 (4%)	0	
Fecal incontinence (preexisting prior to surgery)	0	1 (11%)	
Constipation and pain	1 (4%)	0	
Constipation and fecal incontinence (preexisting prior to surgery)	0	1 (11%)	
Perianal dermatitis	2 (7%)	0	
None	24 (85%)	7 (78%)	

FIA, fistula-in-ano.

## 4. Discussion

We present the first study on non-cutting seton placement in intra- and transsphincteric FIA of infancy and childhood, which is based on retrospective data from a single center. This approach is adapted from adult techniques in order to spare the sphincter complex and reduce postoperative sequela. As classical surgical options like fistulotomy, fistulectomy, and cutting seton are insufficient in anal sphincter protection and drainage, loose seton was proposed in adult therapy (6, 7) and has been evolved to combined options (8). Dekker et al. summarized treatment options of FIA in adults in 2021 and stated that in cases of high FIA and complex or highly inflammatory findings, 90% of the participating surgeons currently use non-cutting seton in the Netherlands as state of the art (9). Removal of seton was observable between 6 and 12 weeks, when epithelialization of the fistulous tract had been achieved.

Seton placement as a therapeutic option in pediatric FIA has rarely been discussed in the literature. Ikeda et al. were able to publish the first study presenting the outcome of FIA patients after cutting seton placement, which is considered the largest series on cutting seton placement in children (10). Of the FIA patients, 97% recovered under therapy with seton. Inoue et al. presented the long-term follow-up of these patients in 2014 and confirmed that fecal incontinence could be excluded as a relevant sequela with a mean follow-up time of 50 months (11). Conclusively, they confirmed a decreased recurrence rate and treatment duration after cutting seton placement in 36 FIA patients aged <1 year (10). Direct comparison to non-cutting techniques should be made with caution and might only be completely possible if the scientific discussion is focusing on infancy/childhood data on pain (postsurgically and after tightening of the seton to cut further), quality of life, and long-term follow-up of fecal incontinence (>40 years of age). Turkyilmaz et al. are to our knowledge the only study group reporting on non-cutting seton placement in FIA in a case presentation of three children (2). Neither recurrence of FIA nor fecal incontinence occurred as sequela. They do, however, not report a time point of healing.

We observe

• A relatively low recurrence rate of 7% with only one patient with recurrent FIA after seton removal (2%), which reflects study outcomes of the so far singular review on childhood FIA (3). Recurrence rates after cutting seton placement have been described in up to 15% in childhood and adult studies (2). Non-cutting seton placement might therefore be a relevant therapeutic option in the surgical treatment of FIA patients.
• No differences between age groups concerning outcome after seton placement, even if the uneven distribution of subgroups has to be acknowledged as a limitation of the study (*n* = 33 vs. 9 patients). Novotny et al. associated an increased recurrence rate with increasing age, regardless of the therapeutic approach (12), which contradicts our results. However, the bias of this uneven distribution regarding only nine patients in the age group of children impedes to draw strong conclusions.
• Fecal incontinence not to be a complication in long-term outcome with a median follow-up time of 47 months, as it was not seen in otherwise healthy infants and children in our population. Studies on adult FIA treatment confirm higher rates of fecal incontinence after cutting seton placement (13, 14), which might probably also be the case after decades in discussed populations of infants with FIA and might not have been recorded in previous studies (11).
• Hints to postsurgical perianal dermatitis and childhood constipation. An increasing number of sequelae with increasing age might additionally prompt for further investigations.

However, we propose to evaluate the following two aspects in further studies, as these might show a substantial influence on postsurgical outcome. To our knowledge, these have not yet been discussed in literature:

Duration of a placed non-cutting seton is not determined so far. We were able to observe a therapeutic success rate of 93% in FIA patients with a median duration of 4.6 months of the placed seton, which was not associated with outcome variables. Inoue et al. described a median healing time of 6.3 ± 4.0 weeks after seton placement compared to a healing time of up to 4 months under conservative options. We assume the median duration of non-cutting seton to be too long in our population and have currently established a prospective, randomized controlled trial as a follow-up study, comparing outcome variables in infants with FIA and non-cutting seton placement for 4 and 12 weeks (registered with clinicaltrails.gov, Identifier NCT05666609). Even in adult treatment, duration of the placed seton is not determined so far and is depending on individual decision (7).

Influence of perioperative antibiotic treatment remains elusive. Afşarlar et al. proposed that the development of FIA after a perianal abscess could be significantly reduced with additional antibiotic therapy (15). However, this is controversially discussed (16) and scientific discussion is lacking data on recurrence of FIA.

Based on the retrospective nature of the study, the long observational period, and the small sample size, limitations to the discussed results and proposed conclusions are certainly given. This is especially noteworthy regarding the comparison of age groups within this study. Based on the small sample sizes of the subgroups and the uneven distribution of sample sizes, statistical assessment must be interpreted with caution. However, a separate analysis of FIA within different age groups should be conducted consequently in further studies. We estimate these findings as a basis for further scientific discussions and see the need for prospective, well-planned, enlarged population studies. These should combine a pediatric surgical approach with the coloproctological knowledge to assess options.

Conclusively, we estimate non-cutting seton placement as a valuable surgical option in the treatment of intra- or transsphincteric FIA in infants and children. This option seems to combine benefits of the surgical approach with low incidence of recurrence while avoiding the destruction of the sphincter.

## Data Availability

The raw data supporting the conclusions of this article will be made available by the authors, without undue reservation.
